# Automated External Defibrillator pad placement pictograms in Japan: sex representation in publicly available product information

**DOI:** 10.1016/j.resplu.2026.101417

**Published:** 2026-07-16

**Authors:** Kentaro Omatsu, Hinata Abe, Takahiro Sakai, Aika Takahashi, Wataru Suzuki, Hiromu Shouji, Taiki Hoshino, Yutaka Takei

**Affiliations:** a Department of Emergency Medical Sciences, Faculty of Medical Technology, Niigata University of Health and Welfare, Niigata, Japan; b Graduate School of Health and Welfare, Niigata University of Health and Welfare, Niigata, Japan

We read with great interest the article by Fijačko et al., which highlighted that instructional pictograms on automated external defibrillators (AEDs), pad packages, and pads overwhelmingly depict male bodies.^1^ We would like to add a Japanese perspective to this discussion.

Japan has implemented public access defibrillation (PAD) since 2004, and approximately 1.5 million AEDs had been sold domestically by 2022.^2^ Despite this widespread availability, sex disparities in PAD persist, including lower rates of AED pad placement among women with out-of-hospital cardiac arrest in public locations and sex- and age-related differences in PAD use and outcomes.^3^, ^4^ The design of visual instructions provided to lay rescuers may be one contributing factor.

Using a classification framework similar to that of Fijačko et al., we audited AED pad placement pictograms in Japan. We reviewed all 23 AED models from eight manufacturers approved for distribution in Japan as of 12 June 2024, as listed on the Ministry of Health, Labour and Welfare website.^5^ Publicly available product information, including official webpages, catalogues, and manuals, was reviewed. Two reviewers independently assessed sex representation in adult AED pad placement pictograms based on visible body characteristics. The audit focused only on sex representation; paediatric pads, paediatric modes, and age-related representations were outside the scope of this analysis. Agreement was complete.

All 23 models displayed male-bodied pictograms for adult pad placement, and no model included female-bodied representation. This proportion was numerically higher than the 62.7% male-only rate reported internationally by Fijačko et al.,^1^ although direct comparison is limited by differences in the materials reviewed. Fijačko et al. evaluated photographs of physical AEDs, pad packages, and pad sets, whereas our audit was based on publicly available product information. Nevertheless, product webpages, catalogues, and manuals are official sources that may be consulted by purchasers, educators, and AED installation managers. Male-only imagery in these materials may reinforce the perception that AEDs are primarily used on male bodies. This is particularly relevant because AED use requires chest exposure and appropriate pad placement around breast tissue.

Our findings corroborate international concerns and suggest that male-centred AED pictogram designs are also prevalent in Japan. Notably, training AEDs were excluded from the original analysis by Fijačko et al.^1^ Given that many lay rescuers first encounter AED instructions during training, inclusive pictograms on training devices and associated educational materials may also be important for shaping rescuer behaviour towards female patients.^6^

Further discussion is warranted regarding sex-inclusive visual instructions for AED use in Japan. The European Resuscitation Council Guidelines 2025 explicitly considered diversity, equality, equity, and inclusion during guideline development and illustrated AED pad positions using both female and male bodies.^7^ Although our findings are based on publicly available product information and should be confirmed by direct examination of physical devices, they indicate that inclusive pictograms should be considered in future AED product design, educational materials, and resuscitation training. Such visual improvements may represent a low-cost and scalable approach to improving the realism of AED guidance and supporting equitable access to PAD.

## Data sharing

The data supporting the findings of this study are available from the corresponding author upon reasonable request.

## CRediT authorship contribution statement

**Kentaro Omatsu:** Conceptualization, Formal analysis, Methodology, Writing – original draft. **Hinata Abe:** Data curation, Investigation, Writing – original draft. **Takahiro Sakai:** Data curation, Investigation, Writing – original draft. **Aika Takahashi:** Data curation, Investigation, Writing – original draft. **Wataru Suzuki:** Data curation, Investigation, Writing – original draft. **Hiromu Shouji:** Data curation, Investigation, Writing – original draft. **Taiki Hoshino:** Data curation, Investigation, Writing – original draft. **Yutaka Takei:** Supervision, Validation, Writing – review & editing.

## Declaration of generative AI and AI-assisted technologies in the writing process

During the preparation of this manuscript, the authors used ChatGPT (OpenAI, Inc.) for English language editing and stylistic refinement. After using this tool, the authors reviewed and edited the content as needed and took full responsibility for the publication’s content.

## Declaration of competing interest

The authors declare no conflicts of interest.
